# Editorial: Neuropsychological and Cognitive-Behavioral Assessment of Neurodegenerative Disease and Rehabilitation Using New Technologies and Virtual Reality

**DOI:** 10.3389/fpsyg.2021.691909

**Published:** 2021-06-21

**Authors:** Marta Matamala-Gomez, Fabrizio Stasolla, Sofia Seinfeld, Alessandro O. Caffò, Domna Banakou, Sara Bottiroli

**Affiliations:** ^1^Department of Human Sciences for Education “Riccardo Massa”, Center for Studies in Communication Sciences “Luigi Anolli” (CESCOM), University of Milano-Bicocca, Milan, Italy; ^2^“Giustino Fortunato” University of Benevento, Benevento, Italy; ^3^Centre de la Imatge i la Tecnologia Multimèdia (CITM), Universitat Politècnica de Catalunya (UPC), Barcelona, Spain; ^4^Department of Educational Sciences, Psychology and Communication, University of Bari, Bari, Italy; ^5^Event Lab, Department of Clinical Psychology and Psychobiology, University of Barcelona, Barcelona, Spain; ^6^Scientific Institute for Research, Hospitalization, and Healthcare (IRCCS), Mondino Foundation, Pavia, Italy

**Keywords:** assessment, rehabilitation, virtual reality, neurodegenerative diseases, technology

Neurodegenerative diseases are characterized by a progressive degeneration of the nervous system and as a consequence, of the brain function (Batista and Pereira, 2016). Such degeneration may affect body movement and brain function, causing an important and progressive decline of the cognitive functions such as memory, thinking, behavior, language, calculation, learning, and emotion capacity (Chekani et al., 2016). The life expectancy of patients presenting neurodegenerative diseases, and the incidence of these has increased over the years, representing one of the most important medical and socio-economic problems of our time (Batista and Pereira, 2016). The rehabilitation process of these patients is long and the assessment periods and follow-up are time consuming for both patients and clinicians. In this regard, the development of new technologies in the last two decades had led to the introduction of different technological devices to enhance the clinical outcomes of conventional clinical interventions, and to facilitate clinical assessments to the clinicians (Moccia et al., 2021). Some of these technological solutions for rehabilitation includes virtual reality, robotic device, non-invasive brain stimulation systems such as transcranial direct current stimulation (TDCS), transcranial magnetic stimulation (TMS), functional electrostimulation techniques, or brain computer interfaces (BCI) (Weiss et al., 2014; Matamala-Gomez et al., 2018; Tamburin et al., 2019). The combination of different technologies for rehabilitation can pave the way to a more holistic rehabilitation intervention for neurological patients presenting multiple deficits because of their clinical condition.

The present Research Topic “Neuropsychological and Cognitive-Behavioral Assessment of Neurodegenerative Disease and Rehabilitation Using New Technologies and Virtual Reality” includes 12 high-quality manuscripts that offer an interesting scenario on these technological advances, as well as new features and approaches for the assessment and rehabilitation of patients with neurodegenerative disorders. Some studies pertained the field of motor and cognitive rehabilitation by using different technologies and approaches. For instance, Chew et al. investigated the effects of priming with tDCS prior to Motor imagery (MI)-BCI training in forty-two patients with chronic stroke presenting moderate to severe upper extremity paresis. The patients were randomized to receive 10 sessions of twenty-min 1 mA real or sham-tDCS before MI-BCI. Results showed that both the real- and sham-tDCS groups improved significantly in UE function with MI-BCI training, with gains continuing up to 4 weeks post-intervention, which were greater in extent in the real-tDCS group.

Cao et al. investigated the sequence effect (SE), which is the reduction in amplitude of step-to-step function which leads to the freezing of gait (FOG) effect in patients with Parkinson's disease (PD), when approaching a destination. Further, the authors also explored the effects of different types of visual cues on destination SE. Thirty-five patients with PD were divided into a freezing group and a non-freezing group. Patients underwent three different conditions while walking: (1) without cues (no-cue condition), (2) wearable laser lights (laser condition), and (3) transverse strips placed on the floor (strip condition). The results from this study showed that patients with PD presenting FOG showed greater destination SE in the no-cue and laser conditions when compared to the patients with PD without FOG. Further, the destination SE was alleviated only by using the transverse strips on the floor. In contrast, transverse strips and wearable laser lights increased the step length. In this line, van der Ham et al. examined technology acceptance for cognitive rehabilitation in a sample of healthcare providers involved in cognitive rehabilitation by using an adapted version of the Technology Acceptance Model (TAM) questionnaire. Results indicated a generally favorable attitude toward the use of digital cognitive rehabilitation and positive responses toward the TAM constructs. This study is interesting as it may stimulate further implementation of digital technologies in cognitive rehabilitation. Similarly, Bottiroli et al. investigated whether Smart Aging, a serious game (SG) platform that generates a 3D virtual reality environment in which users perform a set of screening tasks designed to allow evaluation of global cognition, could differentiate between different types and levels of cognitive impairment in patients with neurodegenerative disease. Ninety-one subjects were involved in this study: healthy older adults (HCs, *n* = 23), patients with single-domain amnesic mild cognitive impairment (aMCI, *n* = 23), patients with single-domain executive Parkinson's disease MCI (PD-MCI, *n* = 20), and patients with mild Alzheimer's disease (mild AD, *n* = 25). Results highlighted significant between-group differences in all the Smart Aging indices, suggesting the validity of this platform as a screening tool for the detection of cognitive impairment in patients with neurodegenerative diseases.

Di Tella et al. aimed at identifying the significant predictors of ecological memory amelioration after the Human Empowerment Aging and Disability (HEAD) virtual rehabilitation program for chronic neurological diseases. The study showed that residual level of cognitive and/or motor functioning is a significant predictor of the treatment success, and an intrinsic relationship between motor and cognitive functions showing the beneficial effects of physical activity on cognitive functions and vice versa. Furthermore, in the manuscript from Pavlidou and Walther the authors proposed that VR can be utilized to restore and improve motor functioning in patients with schizophrenia through VR-mediated motor-cognitive interventions. In this regard, Iosa et al. developed a virtual reality task for upper limb motor rehabilitation, which allows patients, by moving their hand on a virtual canvas, to have the illusion of painting some art masterpieces. The authors conducted two studies, one with 20 healthy subjects, and another with four patients with stroke which performed the experimental task and a control one in which they simply colored the virtual canvas. The results showed that the art condition was performed by healthy subjects with shorter trajectories and with a lower perception of physical demand. The patients with stroke treated with artistic stimuli showed a reduction in the erroneous movements performed orthogonally to the canvas. This study can be relevant for the design of future motor rehabilitation trainings with virtual reality. Mancuso et al. proposed the integration of (i) virtual reality, which immerses the user in a controlled, ecological, and safe environment; and (ii) non-invasive brain stimulation, i.e., transcranial magnetic or electric brain stimulation, which has emerged as a promising cognitive treatment for MCI and Alzheimer's dementia, to cognitive rehabilitation as well as to provide a multimodal stimulation that could enhance cognitive training, resulting in a more efficient rehabilitation. Finally, Burke and Rooney considered the role of VR as a viable platform for the clinical utility of dual-task assessments in an opinion article. The authors highlighted some of the cognitive and neuropsychological considerations to be made when using VR for dual-task assessments in neurodegenerative and neurological conditions.

Robotics are also widely used for motor and cognitive rehabilitation purposes. In this field, Kang et al. aimed at investigating the effect of a Smart Glove Training (SGT) for upper-extremity rehabilitation in patients with sub-acute stroke. The authors conducted a randomized control trial enrolling 23 patients with sub-acute stroke and observed a decrease in upper-extremity impairment after a 2-week of smart glove training period. Similarly, Aprile et al. used a robotic motor/cognitive rehabilitation program in order to enhance cognition in patients with stroke. In this pilot study, patients received an upper limb rehabilitation program consisting in a set of three robots and one sensor-based device comprising both motor and cognitive exercises. Patients underwent 30 rehabilitation sessions, each session lasting 45 minutes, 5 days a week. Results showed that patients improved in all the investigated cognitive domains, as measured by the selected cognitive assessment scales, suggesting that robotic technology can be used to combine motor and cognitive exercises in a unique treatment session. Further, some perspective and opinion articles proposed interesting suggestions and considerations for the future use of VR in the field of assessment and rehabilitation. In particular, Burke and Rooney considered the role of VR as a viable platform for the clinical utility of dual-task assessments. The authors highlighted some of the cognitive and neuropsychological considerations necessary when using VR for dual-task assessments in neurodegenerative and neurological conditions. Mancuso et al. provided a brief review of current evidence regarding the benefits of non-invasive technologies (VR and TMS) on MCI cognitive rehabilitation. They also proposed an integrated intervention approach consisting of VR-based cognitive training and neural stimulation by means of TMS that should act on both a neural-cognitive and behavioral-cognitive, resulting in a more efficient rehabilitation for MCI. Finally, Han et al. introduced a protocol for a randomized controlled trial consisting in the use a novel therapy – known as Remote Ischemic Conditioning (RIC) therapy (designed to protect vital organs from severe lethal ischemic injury by blockage of transient sublethal blood flow to non-vital organs) combined with an exercise (E) therapy — for acute ischemic stroke patients. Overall, this Research Topic aimed to integrate some of the novel information regarding the use of newly developed technologies, robotics, and virtual reality systems for neuropsychological assessment and rehabilitation applied to patients with neurodegenerative diseases. We would like that the manuscripts of this research Topic should — at least in part — shed light on the understanding on how to apply such new technologies to the treatment, monitoring or assessment of neurological patients.

## Author Contributions

MM-G and SB drafted the manuscript. SS, DB, FS, and AC critically revised the manuscript for important intellectual content. All authors conceived, designed the manuscript, and approved the final version of the manuscript.

## Conflict of Interest

The authors declare that the research was conducted in the absence of any commercial or financial relationships that could be construed as a potential conflict of interest.
